# Proximate, Antinutritional, Microbial, and Sensory Acceptability of Bread Formulated from Wheat (*Triticum aestivum*) and Amaranth (*Amaranthus caudatus*)

**DOI:** 10.1155/2020/9429584

**Published:** 2020-11-01

**Authors:** Aemiro Tadesse Zula, Dagim Alemayehu Ayele, Woinshet Abera Egigayhu

**Affiliations:** ^1^School of Nutrition, Food Science and Technology, Center of Excelence in Human Nutrition, Hawassa University, Ethiopia; ^2^Center of Food Science and Nutrition, Addis Ababa University, Ethiopia

## Abstract

**Background:**

Breads are made throughout the world. Bread can be prepared from cereal like wheat, maize, and rice. Nowadays, gluten intolerance, requirement of healthy, and nutritious products have increased and interests towards underutilized crops have also been increasing with the aim of improving global food security and to ease an adverse effect of climate changes. Amaranth is one of nutritionally balanced and naturally grown underutilized crops, but it is mainly considered weed in Africa including Ethiopia.

**Method:**

The aim of the study is to develop bread from wheat and Amaranthus and to evaluate proximate composition, antinutritional, microbial, and sensory acceptability of bread. The experiment contained 100% wheat as control and four blending proportions (90% wheat and 10% amaranth, 80% wheat and 20% amaranth, 70% wheat and 30% amaranth, and 60% wheat and 40% amaranth). A complete randomized design is used for proximate composition, antinutritional, and microbial data analysis whereas a randomized complete block design with three replications was applied for sensory acceptability. SAS for windows version 9 was used for data analysis.

**Result:**

The study revealed that moisture, protein, fat, fiber, and antinutritional content were increased as Amaranthus concentration is increased from 10% to 40%. However, carbohydrate, microbial load, and sensory acceptability were decreased. But the gross energy is constant.

**Conclusion:**

From the study, it can be concluded that beside the good nutritional profile of Amaranthus, it has antinutritional content which needs to limit the concentration of Amaranthus in blending with other grains during product development.

## 1. Introduction

Bread is popular worldwide, and it can be prepared from cereal like wheat, maize, and rice. Nowadays, needs for nutritious products is increasing [1]. Similarly, interests towards underutilized crops have also been increasing with the aim of improving global food and nutrition security. Amaranth is one of nutritionally balanced and naturally grown underutilized crops [2], but it is mainly considered weed in Africa including Ethiopia. Thus, engaging in Amaranth cultivation and appreciation for consumption could be valuable for reducing existed both food and nutrition insecurities in developing countries like Ethiopia.

The consumption of bread from wheat is popular. But the limited nutritional profile of wheat is an alarm to think for other cereal which is good in nutritional profile so as to compliment it with wheat in bread production [3]. According to (Ikram et al., 2010), Amaranthus has carbohydrate (48–69%), protein (12–18%), and fat (5–8%). It has also high concentration of limiting amino acids like lysine (0.747 g) and tryptophan (0.181 g) [4] with numerous benefits. Beside all these, it is also relatively good in sulfur-containing amino acids which are limited in the pulse crops at the normal circumstance high amount of iron, zinc, and calcium [5].

Amaranthus is known in Ethiopia specifically in south and south west parts, but limited concern has been given to the crop. However, in some areas, it is used to prepare local beverage known as “Chaqa,” porridge, pancake-like bread (injera), bread, borde, *kitta* (unleavened bread), and atmit, though bread from wheat and Amaranthus could be good for nutritional profile of bread as it provides energy, vitamins, and minerals.

## 2. Material and Methods

### 2.1. Sources and Preparation of Materials

The raw materials for preparation of bread were wheat and Amaranthus. Wheat was obtained from Hawassa local market, and Amaranthus was obtained from Gamo zone (Arba Minch). The grains were sorted, and extraneous material was removed then washed, cleaned, and sun dried. Both the dried whole wheat and Amaranthus were later milled using cyclone mill and sieved into fine flour of uniform particle size by passing them through a 0.5 mm mesh screen.

### 2.2. Preparation of Wheat Flour

The extraneous matter was removed from wheat, and then, the grain was washed, cleaned using tap water, drained, sundried, and milled using a cyclone mill to pass through a 0.5 mm mesh screen so as to get the flour. The milled grain was then packed by polyethylene bag and finally stored at room temperature.

### 2.3. Preparation of Amaranthus Flour

The Amaranthus grain was cleaned from extraneous matter and soaked in steam water for 12 h with 1 : 3 (*w*/*v*) concentration to ensure effective removal of antinutrients [6]. The initial temperature of steam was around 70°C held for 10minutes, and the water was changed at a six-hour interval. The Amaranthus grain was sundried and milled using a cyclone mill (Tecator AB, Haganas, Sweden) to pass through a 0.5 mm mesh screen and filled in polyethylene bags. After getting the flours of Amaranthus and wheat, it was mixed according to the formulation (Table 1).

### 2.4. Preparation of Bread

Preparation of dough for bread was done by mixing 1% iodized salt, composite flour, yeast, and water. After mixing all ingredients, composite flour was kneaded until it becomes soft, smooth, and stiff and kept for two and half hours for rising (fermentation). Preheated local clay griddle (*Mitad*) was used for baking, and the leaf of enset (*Ensete ventricosum*) was used for wrapping the dough to be baked. The baking was continued until a brown color appeared (which will take about 25 minutes at 150^o^c). The bread which was prepared was kept at room temperature to cool down, wrapped using polyethylene bags.

### 2.5. Chemical Composition Analysis

The proximate composition of bread was determined according to [7]. The moisture content was determined using official method 934.01, ash content was determined using official method of 923.03, crude fat content was determined using official method of 920.39, crude protein was determined using official method of 981.10, crude fiber was determined by [8], and total carbohydrate was determined by difference method. Condensed tannin and phytate contents were determined by using the method used by [9]. The phytate content was calculated by dividing the measured value of phytic acid by molecular weight (240) of phytic acid.

### 2.6. Microbial Analysis of Bread

Total mold, yeast, and bacteria counts were carried out on bread samples after 2-day room temperature storage using the procedure of [10]. Bread samples were taken aseptically and homogenized in 99 ml sterile peptone water 0.1% in a blender for about 2 minutes, and serial dilutions were made. Dilution of 0.1 ml was spread plated in sterile Petri dishes, the stomacher dilutions of 10^−1^, 10^−2^, 10^−3^, 10^−4^, and 10^−5^ were prepared by using 9 ml peptone water tubes and plate count agar (PCA) with the addition of chloramphenicol and incubated at 25°C for 5 days for mold and yeast count, and molten plate count agar (PCA) was used and incubated for 48 hours at 35°C for total bacterial count. Counts of visible colonies by using colony counter were made and expressed as log CFU/g of the original sample.

### 2.7. Sensory Evaluation of Breads

The bread samples were coded with three digit numbers, and randomly, the samples were given to randomly presented panelists in a random order. The sensory evaluation was carried out using a five-point hedonic scale (1 = dislike very much, 2 = dislike, 3 = neither like nor dislike, 4 = like, and 5 = like very much) in terms of color, taste, aroma, texture, and overall acceptability with 20 panelists in triplicate.

### 2.8. Experimental Design

Treatments with blending at different proportions of wheat and amaranth (90 : 10, 80 : 20 and 70 : 30, 60 : 40) and 100% wheat (control) were used to asses chemical composition, microbial load, and sensory acceptability. A complete randomized design was used for chemical composition and microbial load analysis whereas a randomized complete block design (RCBD) was used for sensory acceptability analysis.

### 2.9. Data Analysis

One-way analysis of variance using SAS software version 9 was used for data analysis. The mean separation was done using Tukey's HSD test at *p* < 0.05.

## 3. Result and Discussion

### 3.1. Proximate Compositions

The proximate composition of bread formulated from wheat and Amaranthus is presented in (Table 2). Moisture content of bread is varied from 7.18 to 7.71. Bread made from 20%, 30%, and 40% of Amaranthus had higher (*p* < 0.05) moisture content as compared to control and 10% Amaranthus-blended bread. The study revealed that the moisture content was increased as Amaranthus concentration is increased. The higher moisture content of bread made from higher Amaranthus concentration is might be due to high water absorption capacity of Amaranthus as reported by [11].

The protein content of bread is varied from 8.17 to 9.96. Bread made from 30% and 40% Amaranthus had higher (*p* < 0.05) protein content as compared to bread made from 20% and 10% Amaranthus and control (100% wheat). The higher protein content is from bread made from 40% of Amaranthus, and Amaranthus concentration increases as the protein content increases. The increase in protein content might be due to the fact that Amaranthus has high protein content as compared to wheat [12].

The fat content is varied from 3.95 to 4.94. Bread made from 10%, 20%, 30%, and 40% Amaranthus had higher (*p* < 0.05) fat content as compared to control (10% wheat). The study showed, as amaranthus concentration increases from 10% to 40%, the fat content was increased. The increase in fat content as Amaranthus increased is because Amaranthus has higher nutritional profile as compared to wheat and other cereals as reported by [13].

The ash content of bread is varied from 1.36 to 1.99. Bread made from 20%, 30%, and 40% of Amaranthus had higher (*p* < 0.05) ash content as compared to bread made from 10% Amaranthus and control (100% wheat). The increase in ash content might be due to Amaranthus having higher mineral content than wheat.

The fiber content is varied from 1.86 to 2.99. The fiber content is slightly increased as Amaranthus concentration is increased from 10% to 40%. However, bread made from 30% and 40% Amaranthus had significantly higher (*p* < 0.05) fiber content as compared to control (100% wheat), 10%, and 20%. The increase in fiber content as Amaranthus increased is because Amaranthus has good nutritional profile and higher fiber content as it is a very fine cereal as compared to wheat [12].

The carbohydrate is varied from 77.48 to 73.41. The carbohydrate is slightly decreased as Amaranthus concentration is increased from 10% to 40%. The decrease in carbohydrate as Amaranthus increased is because of the increase in moisture, protein, fat, ash, and fiber.

The gross energy is varied from 378.15 to 377.94. The energy is insignificantly decreased (*p* > 0.05). The increase in gross energy might be due to the increase in carbohydrate as Amaranthus concentration is increased.

### 3.2. Antinutritional Content

**Figure 1:**
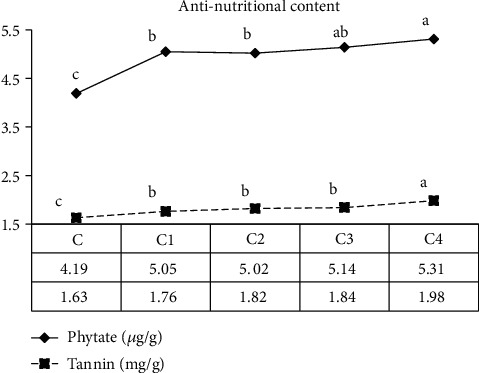
Effect of blending ratio on antinutritional content of wheat-amaranth-based bread.

The antinutritional content of bread formulated from wheat and Amaranthus is presented in (Figure 1). The antinutritional (phytate and tannin) content of bread is varied from 4.19 to 5.31 and 1.63 to 1.98, respectively. Bread made from 30% and 40% of Amaranthus had similar (*p* > 0.05) phytate content, and similarly, bread made from 10%, 20%, and 30% of Amaranthus had also similar (*p* > 0.05) phytate content, but they had higher (*p* < 0.05) phytate content as compared to control (100% wheat). Bread made from 10%, 20%, and 30% of Amaranthus had similar (*p* > 0.05) tannin content, but they had higher (*p* < 0.05) tannin content compared to control (100% wheat) and lower (*p* < 0.05) tannin content as compared to bread made from 40% of Amaranthus.

In general, the study revealed that the antinutritional content of bread is increased as Amaranthus content is increased from 10% to 40%. The increase in phytate and tannin content as Amaranthus concentration increased is because as reported by [13] Amaranthus had high antinutritional content.

### 3.3. Microbial Load

**Figure 2:**
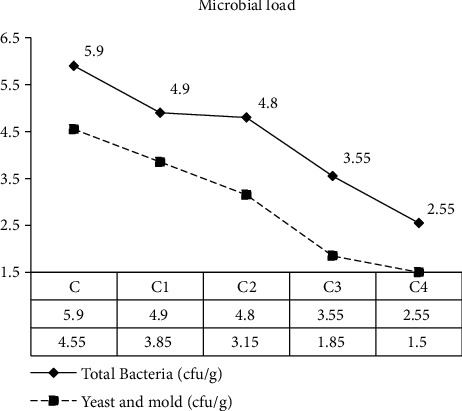
Effect of blending ratio on microbial load (total plate count, mold, and yeast) of wheat-amaranth bread.

The microbial load of bread is shown in (Figure 2). Total bacteria of bread were varied from 5.9 cfu/g to 2.55 cfu/g. Bread made from 40% of Amaranthus and 60% of wheat had lower total bacteria as compared to control (100% wheat), and the study indicates that as Amaranthus concentration increases, the total bacteria count was decreased significantly (*p* < 0.05). The decrease in total bacteria as Amaranthus increased might be due to Amaranthus being soaked in steam water for 24 h; this indicates there was lower microbial growth for steam water products as steam slows down the microbial growth. Generally, when comparing with microbiological standards of blended foods, total bacteria count has 10^3^ to 10^5^ cfu/g which was still within an acceptable value. It was known that total plate count values for cereal and legume-based products exceeding 10^6^ CFU/g are considered microbiologically unsafe [14]. From this investigation, none of the treatment was exceeded over 10^6^ CFU/g.

Both yeast and mold count are shown in (Figure 2). The mean value for yeast and mold of bread was varied from 4.55 cfu/g to 1.55 cfu/g. Bread made from 40% of Amaranthus and 60% of wheat had lower yeast and mold as compared to control (100% wheat, 90%: 10, 80%: 20, 70%: 30), and the study indicated as Amaranthus concentration increases, mold and yeast count are decreased significantly (*p* < 0.05). The decrease in mold and yeast as Amaranthus increased might also be due to Amaranthus being soaked in steam water for 24 h; this indicates there was lower microbial growth for steam water products as steam slows down the microbial growth.

The sensory acceptability of breads is shown in (Table 3). The color, taste, aroma, texture, and overall acceptability of bread were varied from 3.16 to 4.64, 3.14 to 4.5, 3.28 to 4.45, 2.68 to 4.52, and 3.4 to 4.61, respectively. Bread made from 100% wheat had higher sensory acceptability as compared to bread made from wheat substituted with 10%, 20%, 30%, and 40% of Amaranthus. The study showed the color, taste, aroma, texture, and overall acceptability were decreased significantly (*p* < 0.05) as Amaranthus concentration is increased. The decrease in sensory acceptability might be due to the fact that Amaranthus is dark red in color and not well adapted by a consumer as people adapted the white color of bread. According to Lorenz (1981), Amaranthus-supplemented product has got nutty flavor which is not acceptable by the panel in terms of taste and aroma as the concentration increased. The decrease in texture might be due to the water absorption capacity of Amaranthus. According to [15], the water absorption can be increased as Amaranthus concentration increased in injera production with *teff*.

## 4. Conclusion

In conclusion, proximate composition (moisture, protein, fat, ash, and fiber) and antinutritional profile (phytate and tannin content) of bread become high toward the increment of Amaranthus. However, carbohydrate, gross energy, and microbial load (total bacteria, mold and yeast count) were lowered as Amaranthus concentration increased and treatments were within an acceptable range of microbial load below 10^6^ CFU/g. From the study in all treatment as the Amaranthus concentration increases, the color, taste, aroma, texture, and overall acceptance were decreased. Considering the result obtained, the Amaranthus substitution up to 40% of soaking had lower acceptability. However, it is evident that all treatments are within acceptable sensory characteristics.

## Figures and Tables

**Table 1:** Formulation of wheat and Amaranthus flour.

Composite flour	C	C1	C2	C3	C4
Wheat flour	100%	90%	80%	70%	60%
Amaranthus flour	_	10%	20%	30%	40%

C is control (100% wheat), C1 is 90% wheat and 10% Amaranthus, C2 is 80% wheat and 20% Amaranthus, C3 is 70% wheat and 30% Amaranthus, and C4 is 60% wheat and 40% Amaranthus.

**Table 2:** Effect of blending ratio on proximate composition of wheat-amaranth bread.

Treatment	Moisture (%)	Protein (%)	Fat (%)	Ash (%)	Fiber (%)	Carbohydrate	Energy
C	7.18 ± 0.02^b^	8.17 ± 0.13^b^	3.95 ± 0.01^c^	1.36 ± 0.00^d^	1.86 ± 0.01^c^	77.48 ± 0.14^a^	378.15 ± 0.38^a^
C1	7.25 ± 0.03^b^	8.23 ± 0.35^b^	4.12 ± 0.29^b^	1.35 ± 0.01^d^	1.95 ± 0.01^c^	77.10 ± 0.24^a^	378.46 ± 0.29^a^
C2	7.67 ± 0.14^a^	9.34 ± 0.34^ab^	4.42 ± 0.08^ab^	1.46 ± 0.27^c^	2.16 ± 0.27^b^	74.95 ± 0.18^b^	376.94 ± 0.51^a^
C3	7.86 ± 0.11^a^	9.83 ± 0.13^a^	4.66 ± 0.01^ab^	1.78 ± 0.28^b^	2.78 ± 0.28^a^	73.09 ± 0.28^c^	376.62 ± 0.42^a^
C4	7.71 ± 0.02^a^	9.96 ± 0.12^a^	4.94 ± 0.02^a^	1.99 ± 0.02^a^	2.99 ± 0.02^a^	73.41 ± 0.15^c^	377.94 ± 0.35^a^

C is control (100% wheat), C1 is 90% wheat and 10% Amaranthus, C2 is 80% wheat and 20% Amaranthus, C3 is 70% wheat and 30% Amaranthus, and C4 is 60% wheat and 40% Amaranthus. Means followed by different superscript letters across the column indicate significant difference at *p* < 0.05.

**Table 3:** Effect of blending ratio on sensory acceptability of wheat-amaranth bread.

Treatment acceptability	Color	Taste	Aroma	Texture	Overall
C	4.64 ± 0.48^a^	4.50 ± 0.55^a^	4.45 ± 0.50^a^	4.52 ± 0.50^a^	4.61 ± 0.49^a^
C1	4.11 ± 0.59^b^	4.07 ± 0.46^b^	3.88 ± 0.70^b^	3.83 ± 0.85^b^	4.07 ± 0.60^b^
C2	3.66 ± 0.52^c^	3.45 ± 0.50^c^	3.61 ± 0.62^bc^	2.92 ± 0.51^c^	3.78 ± 0.56^c^
C3	3.19 ± 0.55^d^	3.35 ± 0.48^cd^	3.48 ± 0.62^c^	2.71 ± 0.55^cd^	3.40 ± 0.54^d^
C4	3.16 ± 0.53^d^	3.14 ± 0.35^d^	3.28 ± 0.70^d^	2.68 ± 0.47^cd^	3.40 ± 0.49^d^

C is control (100% wheat), C1 is 90% wheat and 10% Amaranthus, C2 is 80% wheat and 20% Amaranthus, C3 is 70% wheat and 30% Amaranthus, and C4 is 60% wheat and 40% Amaranthus. Means followed by different superscript letters across the column indicate significant difference at *p* < 0.05.

## Data Availability

The data used and/or analyzed in the study are available from the corresponding author on reasonable request.
